# Set2‐mediated H3K36 methylation states redundantly repress the production of antisense transcripts: role in transcription regulation

**DOI:** 10.1002/2211-5463.13226

**Published:** 2021-06-28

**Authors:** Yu‐Chao Mei, Jiangpeng Feng, Fei He, Yu‐Min Li, Yafei Liu, Feng Li, Yu Chen, Hai‐Ning Du

**Affiliations:** ^1^ Hubei Key Laboratory of Cell Homeostasis College of Life Sciences RNA Institute Wuhan University China; ^2^ State Key Laboratory of Virology College of Life Sciences RNA Institute Wuhan University China; ^3^ State Key Laboratory of Biocatalysis and Enzyme Engineering School of Life Sciences Hubei University Wuhan China; ^4^ School of Basic Medical Sciences Wuhan University China

**Keywords:** antisense transcription, H3K36 methylation, Set2

## Abstract

Methyltransferase Set2‐mediated methylation of histone H3 lysine 36 (H3K36), which involves the addition of up to three methyl groups at this site, has been demonstrated to function in many chromatin‐coupled events. The methylation of H3K36 is known to recruit different chromatin effector proteins, affecting transcription, mRNA splicing and DNA repair. In this study, we engineered two yeast *set2* mutants that lack H3K36 mono/dimethylation (H3K36me1/2) and trimethylation (H3K36me3), respectively, and characterized their roles in the production of antisense transcripts under nutrient‐rich conditions. Using our new bioinformatics identification pipeline analysis, we are able to identify a larger number of antisense transcripts in *set2∆* cells than has been published previously. We further show that H3K36me1/2 or H3K36me3 redundantly repressed the production of antisense transcripts. Moreover, gene ontology (GO) analysis implies that H3K36me3‐mediated antisense transcription might play a role in DNA replication and DNA damage repair, which is independent of regulation of the corresponding sense gene expression. Overall, our results validate a coregulatory mechanism of different H3K36 methylation states, particularly in the repression of antisense transcription.

## Graphical abstract

Using a new bioinformatics identification pipeline analysis, thousands of antisense transcripts are identified in Set2‐deficient cells under nutrient‐rich conditions, which are transcribed from the opposite strand of sense genes and usually silenced in wild‐type cells. By engineering two single‐site mutations, we show that H3K36me1/2 or H3K36me3 redundantly repressed antisense transcripts, which suggests a coregulatory mechanism of different H3K36 methylation states.

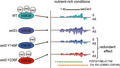

**Abbreviations**
ATE: antisense transcripts enriched
CISs: cryptic initiation sites
CUTs: cryptic unstable transcripts
GO: gene ontology
H3K4: histone H3 lysine 4
H3K36: histone H3 lysine 36
H3K36me1/2: H3K36 mono/dimethylation
H3K36me3: H3K36 trimethylation
NET‐seq: native elongating transcript sequencing
polyA: polyadenylated
RNA-Seq: RNA sequencing
SET: Su(var)3-9, enhancer of zeste and trithorax
SRATs: Set2-repressed antisense transcripts
SUTs: stable unannotated transcripts
WT: wild‐type
XUTs: Xrn1-sensitive unstable transcripts

Antisense transcripts are a class of long noncoding RNAs that originate from the opposite strand of the sense transcripts of protein‐coding genes or non‐protein‐coding genes. Antisense transcripts were discovered in both bacteria [1] and eukaryotes [2]. In the human genome, more than 30% of annotated mRNAs produce antisense transcripts [3]. However, the abundance of antisense transcripts is much lower than that of sense genes due to extensive RNA degradation pathways, which makes them hard to detect. The genomic methods widely developed over the years have allowed researchers to identify numerous novel antisense transcripts and to understand that they generally appear throughout the entire genome of various species [4, 5, 6].

In *Saccharomyces cerevisiae*, antisense transcripts are usually associated with cryptic transcription from cryptic promoters [7]. A large number of cryptic transcription events occur when the transcription complex makes any errors during the transcription process [8, 9, 10, 11]. It has been demonstrated that Set2‐mediated histone H3K36 methylation plays an important role in this process involving mechanisms such as suppressing increased histone acetylation and regulating histone mislocalization, which can inhibit the production of antisense transcripts [12]. Some recent studies have shown that mutations in core proteins associated with transcription processes or chromatin remodelling lead to a change in chromatin structure, and thus, cryptic transcription occurs. For example, a lack of *ISW1* or *IOC4* will cause nucleosome incorrect positioning, making the Rpd3S complex unable to bind to nucleosomes normally, and lead to increase the level of histone acetylation and cryptic transcription [12, 13, 14]. These results imply that Set2‐mediated H3K36 methylation plays an important role in cryptic transcription. Indeed, a novel group of Set2‐repressed antisense transcripts has been identified upon deletion of *SET2* [15]. Recently, DiFiore et al. further revealed roles of different H3K36 methylation states on antisense transcription upon nutrient deprivation [16]. Whether different H3K36 methylation states will impact on the production of antisense transcription genome‐wide under nutrient‐rich conditions remains unclear.

Here, we established two *set2* mutations that specifically lack H3K36me1/2 and H3K36me3. These mutants provide a useful tool to analyse the effect of different methylation states on the production of antisense transcripts, and lead to the identification of a large amount of antisense transcripts repressed by the Set2 protein. Gene expression and GO analyses showed that the expression of these antisense transcripts did not affect the expression of the corresponding sense genes. Moreover, we illustrated that H3K36me1/2 and H3K36me3 methylation showed strong consistency in the expression of antisense transcripts, indicating that a redundant regulatory mechanism occurs. Our study provides further evidence that H3K36 methylation redundantly functions in the production of antisense transcripts under nutrient‐rich conditions.

## Materials and methods

### Yeast strains and plasmids

BY4741 background yeast strains used in this study include wild‐type, *set2∆* expressing pRS415 empty vector (with Leu‐selective marker), *set2∆* expressing *set2*‐Y149F (pRS415 vector) and *set2∆* expressing *set2*‐Y236F (pRS415 vector). Yeast cells were grown at 30 °C in YPD medium (1% yeast extract, 2% peptone and 2% dextrose) or SD medium (0.67% yeast nitrogen base without amino acids, supplemented with appropriate amino acids and 2% glucose). Set2 mutations were generated by the site‐directed mutagenesis method from Invitrogen.

### Western blotting

Western blot analysis was carried out following the protocol as described previously [17]. The following antibodies were used: anti‐Set2 (homemade 1 : 2000), anti‐G6PDH (Sigma, St. Louis, MO, USA, A9521, 1 : 20 000), anti‐H3 (active motif, 39163, 1 : 10 000), anti‐H3K36me1 (ABclonal, Wuhan, China, A2364, 1 : 1000), anti‐H3K36me2 (ABclonal, A2365, 1 : 5000) and anti‐H3K36me3 (ABclonal, A2366, 1 : 3000).

### mRNA extraction and strand‐specific RNA sequencing

Yeast cells were grown overnight in YPD or SD medium at 30 °C, diluted to an OD_600_ of 0.2 and collected when the OD_600_ reached 0.8. Cells were harvested by spinning down at 3000 r.p.m. for 5 min, and then, the cell pellets were washed with 10 mL sterile water twice. Total RNA was extracted with HiPure Yeast RNA Kit (Magen, Guangzhou, China, R4182‐02). The purity and yield of RNAs were examined by NanoDrop One Spectrophotometer (Gene Company). RNA integrity was examined by agarose electrophoresis.

For preparation of mRNA, 20 μg of total RNA was diluted into 50 μL of DEPC water and incubated with 50 μL of mRNA capture beads as described in the product manual (Vazyme, Nanjing, China, N401‐01). Then, the enriched mRNA was used for strand‐specific library construction by the KAPA Stranded RNA Sequencing (RNA‐Seq) Library Preparation Kit (KAPA, KK8401) through the dUTP method. The libraries were used to generate a total of ˜ 6 GB sequencing data from 75‐bp length single‐end reads using a NextSeq CN500 equipment.

### Identification of antisense transcripts

The reads obtained from each sample were mapped to the yeast genome SacCer3 (https://www.yeastgenome.org/) with tophat (version 2.1.1). For each sample, a BAM file was used for subsequent processing. In *SET2* deletion mutants, the BAM file was divided into forward and reverse strands by bedtools (version 2.29.2). The file contained both the reads of the existing annotated transcripts and the antisense reads in the opposite direction. samtools (version 1.10) was used to filter antisense reads of both forward and reverse strands by SAM flags. The bedtools software was used to obtain the reads of intergenic regions. Finally, the antisense reads from the forward strand, the reverse strand and the intergenic region were merged together to obtain an integrated BAM file. Antisense reads were assembled without reference genome annotation in cufflinks (version 2.2.1). The expression levels of antisense transcripts with a *P*‐value < 0.05 were analysed using the gene annotation file of antisense transcripts by Cuffdiff.

### Differentially expressed genes and gene ontology analysis

Data were analysed in the R (version 3.5.0). Differential expression analysis was done in edger (version 3.30.3). Gene ontology analysis was performed by clusterprofiler (version 3.16.0). Adjusted *P*‐value < 0.05 was used for term ranking and selection. All plots and graph were created using ggplot2 in R.

### Structural remodelling

Set2 protein structure was modelled by I‐TASSER (https://zhanglab.ccmb.med.umich.edu/I‐ TASSER/) [18] using the SETD2 crystal structure in Protein Data Bank (PDB, 5JLB) as a template [19]. modeller was used to model different mutations of Set2, and they were visualized in pymol (version 2.4.1) [16].

### Northern blotting

Northern blot was carried out using stranded‐specific DIG‐labelled probes as described previously [20]. Briefly, pairs of DNA primers were designed, which contain T7 promoter on either the sense strand or the antisense strand, and were utilized for PCR amplification to produce about 500‐bp length DNA templates. An amount of 200 ng DNA template was used to generate DIG‐labelled RNA probe in an *in vitro* transcription reaction with HiScribe T7 High Yield RNA Synthesis Kit (NEB, #E2040S) and digoxigenin‐11 labelled UTP (Roche, Cat. No. 12039672910). The primers used to prepare the riboprobes are listed below: *PSP2*‐F: 5’‐TAATACGACTCACTATAGGA GGTGGCGACGATAAAGCTC‐3’; *PSP2*‐R: 5’‐CAAGGAACCCACTTGCTGGT‐3’; *PPN1*‐F: 5’‐TAATACGACTCACTATAGGGATTGCCATCCTGTCGACCT‐3’; *PPN1*‐R: 5’‐TCCGGTCATGTATCTGA‐3’; *SCR1*‐F: 5’‐TAATACGACTCACTAT AGATGGTTTCGGTGGTGG‐3’; and *SCR1*‐R: 5’‐CGCCAAATTAAACCGCCGAA‐3’.

Strand‐specific DIG‐labelled northern blot was carried with 30 μg total RNA. Briefly, the RNA mixed with an equal volume of 2 × RNA loading was denatured at 65 °C for 10 min and allowed to stand on ice for 1 min. Then, it was separated on a 1.2% agarose/formaldehyde (2.2 m) gel running in 1 × MOPS buffer (20 mm MOPS, pH 7.0, 5 mm sodium acetate and 2 mm EDTA in DEPC‐treated water). After separating the RNA, the gel was visualized with ultraviolet light and washed thrice with distilled water to remove formaldehyde. The gel was then rinsed in 20 × SSC (3 m NaCl and 300 mm sodium citrate, pH 7.0) for 15 min twice and transferred to a positive charged nylon membrane. The bolt was cross‐linked under UV for 5 min and prehybridized with 2 mL DIG Easy Hyb Granules (Roche, Roche, Switzerland, Cat. No. 11 796 895 001) at 68 °C for 1 h. The DIG‐labelled RNA probe was added to the buffer after heating at 100 °C for 5 min and incubated at 68 °C overnight. The blots were then washed twice in 2 × SSC and 0.1 × SSC for 15 min. After hybridization and stringency washes, membrane was rinsed briefly for 1‐5 min in washing buffer (0.1 m maleic acid, 0.15 m NaCl, pH 7.5, and 0.3% Tween‐20). The blot was then incubated for 30 min in 10 mL blocking solution and 30 min in 10 mL antibody solution (anti‐digoxigenin‐AP, 1 : 10 000; Roche, Cat. No. 12039672910). After washing twice for 2 × 15 min in washing buffer, the blot was equilibrated in detection buffer (0.1 m Tris/HCl and 0.1 m NaCl, pH 9.5) and CDP‐Star was used to expose the blots in imaging device for 1000 s.

## Results

### Phe/Tyr switch mutations in Set2 generate a tool to distinguish different H3K36me states

In 2003, Xiao et al. demonstrated that the mutation of tyrosine at position 245 to alanine or phenylalanine in SET7/9 would convert the ability of SET7/9 to catalyse the monomethylation of histone H3K4 into dimethylation or trimethylation activity [21]. Protein structural analysis showed that the Phe/Tyr transition at certain sites on some methyltransferases could affect their enzymatic activities [22, 23]. Recently, DiFiore et al. found that the Phe/Tyr switch located in the SET domain of Set2 separates H3K36me states both *in vivo* and *in vitro* [16]. The SET domain, approximately 130 amino acids in length, was named after three *Drosophila* proteins, namely Su(var)3‐9, enhancer of zeste and trithorax, which are characterized by an evolutionarily conserved domain in all eukaryotes [24]. Of note, the SET domain is responsible for the catalytic activity of the SET domain‐containing proteins [24].

**Fig. 1:**
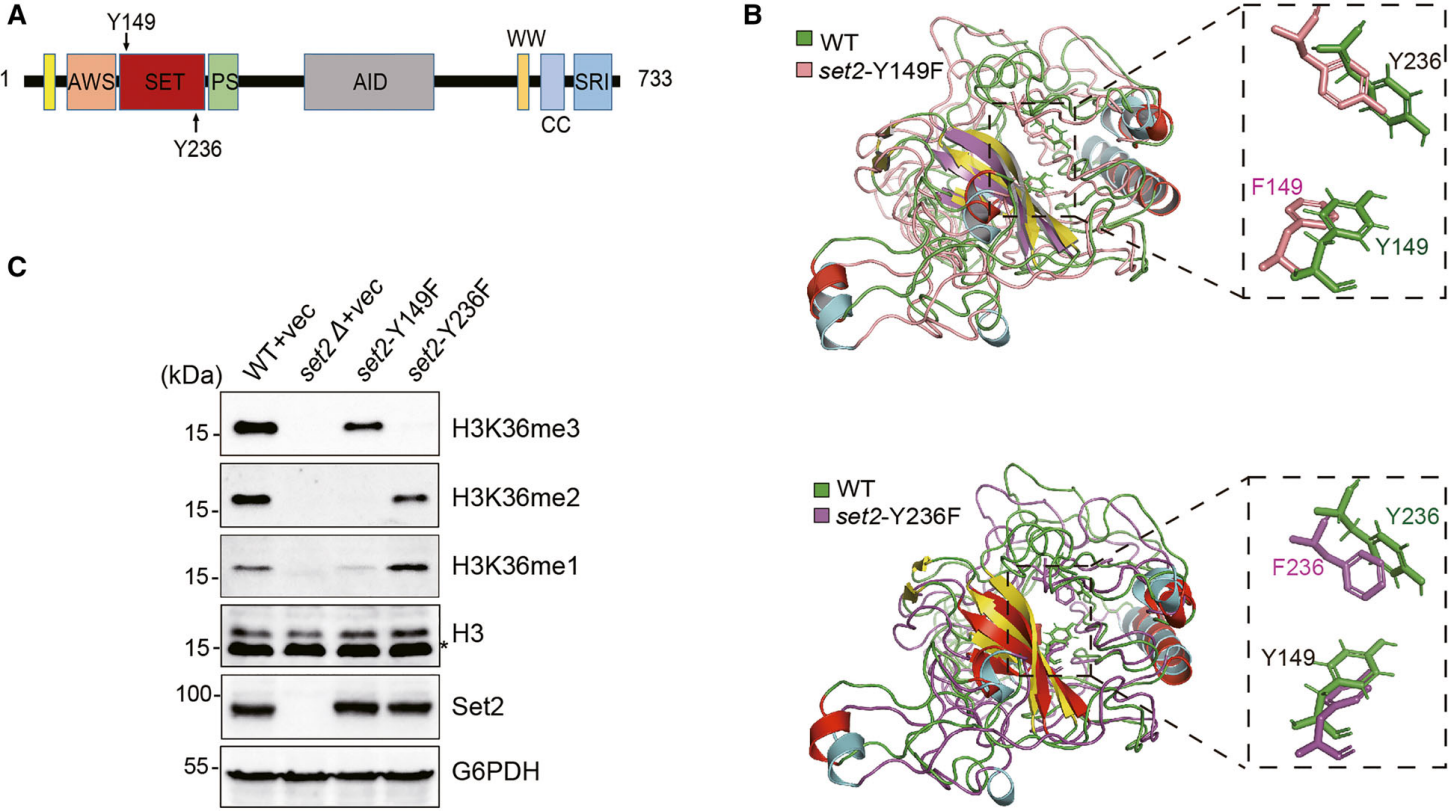
Phe/Tyr switch mutations in Set2 generate a tool to distinguish between different H3K36me states. A, Diagram of the Set2 protein structure with the Y149 and Y236 residues highlighted. AWS, associated with SET, SET, catalytic SET domain; PS, post‐SET; AID, the autoinhibitory domain; CC, coiled‐coiled; SRI, Set2‐Rpb1‐interacting domain. B, Structural comparison of the SET domain of WT Set2 protein (green) with that of the *set2*‐Y149F (pink) or *set2*‐Y236F mutant (purple). The local conformations of Y149 and Y236 that form the Phe/Tyr switch in each structure are highlighted. C, Western blot analysis of the different H3K36 methylation levels in the indicated strains.

Our group independently discovered that engineered Set2 mutants could produce different methylation states of H3K36, which allows us to explore their unique or shared functions. It is noted that the primary sequences of the SET domains between yeast Set2 and human SETD2 proteins are relatively conserved (Fig. S1A). Structural remodelling indicated that the SET domain of yeast Set2 protein is very similar to that of the human SETD2 protein (Fig. S1B). Therefore, the SET domain structure of yeast Set2 protein was utilized to execute structural prediction. Through structural analysis described previously [23], two tyrosine residues (Y149 and Y236) located in the SET domain emerged as key residues impacting H3K36 methylation by altering the SET domain lysine‐binding pocket (Fig. 1A–B). We mutated Y149 and Y236 to phenylalanine, named *set2*‐Y149F and *set2*‐Y236F, respectively, and their catalytic activities were examined *in vivo*. Consistent with previous results [16], the *set2*‐Y149F mutation showed loss of H3K36 dimethylation and decreased H3K36 monomethylation but maintained H3K36me3, whereas the *set2*‐Y236F mutation showed loss of H3K36me3 but maintained H3K36me1/2 (Fig. 1C). Thus, these two Tyr/Phe amino acid switch mutations provide us with a good tool to study the roles of different H3K36 methylation levels in producing antisense transcription.

### Identification of antisense transcripts repressed by Set2

**Fig. 2:**
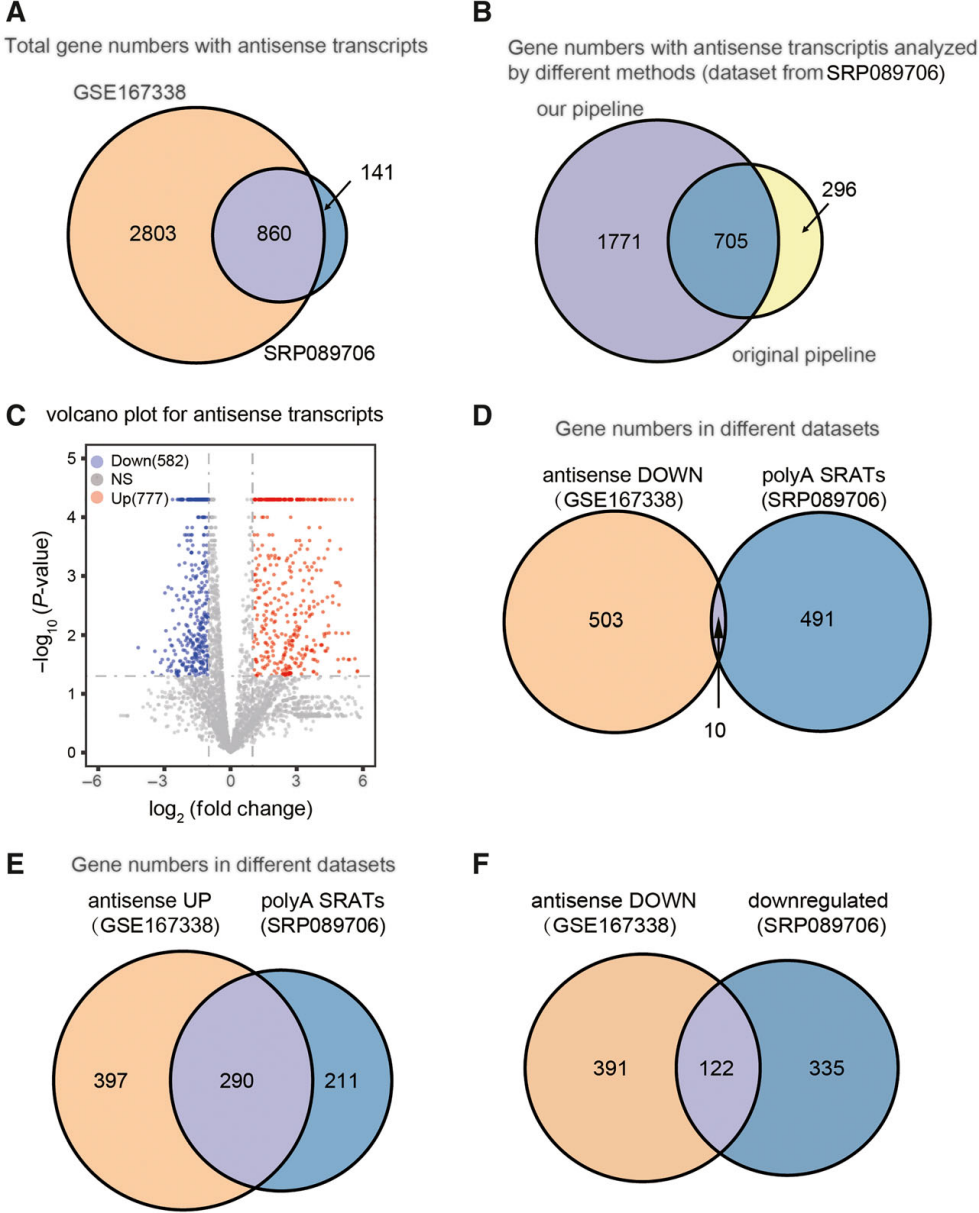
Identification of antisense transcripts repressed by Set2. A, Venn diagram comparison between antisense transcript‐enriched genes in our study (GSE167338) vs. total SRAT‐associated genes (SRP089706) identified previously upon deletion of *SET2*. B, Venn diagram showing the gene numbers with antisense transcripts identified in the dataset SRP089706 that are analysed by different bioinformatics analysis. C, Volcano plot showing the expression profile of the candidate antisense transcripts in the *set2Δ* mutant. Red dots represent significantly upregulated antisense transcripts, and blue dots represent significantly downregulated antisense transcripts. D, E, F, Venn diagram comparison between antisense transcripts (from dataset GSE167338) vs. polyA SRATs (from dataset SRP089706). antisense_down, the downregulated antisense transcript upon loss of Set2 (panel D); antisense_up, the upregulated antisense transcripts upon loss of Set2 (panel E); downregulated: antisense transcripts identified from the published dataset by our experimental pipeline (panel F).

To identify the antisense transcripts produced upon deletion of *SET2*, *set2*‐Y149F or *set2*‐Y236F mutations, total RNA was extracted from the wild‐type (WT) yeast strain and the mutant strains grown in nutrient‐rich medium, and enriched mRNA with polyadenylated (polyA) tails was used to prepare a strand‐specific library. Strand‐specific RNA‐seq was performed. Using our new pipeline, we were able to identify more antisense transcripts than a previous study [15]. It has been shown that 6670 antisense transcripts (GEO: GSE167338) were generated in total 3663 corresponding sense genes upon deletion of *SET2* in our dataset, whereas 1179 antisense transcripts (SRA: SRP089706) were found from total 1001 corresponding sense genes published previously [15] (Fig. 2A). The antisense transcripts enriched (ATE) genes shown in our experiment covered 85.9% of Set2‐repressed candidate antisense transcript (SRAT) genes that were found previously [15] (Fig. 2A). By using our experimental pipeline to reanalyse the previous published dataset (SRP089706) [15], we were able to identify 2476 SRAT‐associated sense genes in *set2Δ* cells, which is 2.4‐fold more than the 1001 SRAT‐associated sense genes that were identified using their pipeline (Fig. 2B). Since the previous study set a selected threshold with twofold changes and a *P*‐value less than 0.05 as parameters to pare down polyA SRAT genes, we decided to use the same parameters to further analyse the ATE genes in our dataset. Based on these criteria, we eventually identified 777 upregulated antisense transcripts and 582 downregulated antisense transcripts (Fig. 2C). In contrast, they found a list of 501 polyA SRAT‐enriched sense genes that are only associated with upregulated antisense transcripts upon loss of Set2 [15]. Among these selected antisense transcripts, there was only 2% overlap of corresponding sense genes between the downregulated antisense transcripts in our data and polyA SRATs reported previously [15] (Fig. 2D), whereas approximately 58% upregulated antisense polyA SRAT‐associated sense genes were found in our data, suggesting the suppressive role of the Set2 protein (Fig. 2E). Intriguingly, 457 downregulated antisense transcripts were identified in the published dataset using our experimental pipeline, in which only 27% overlaps with our results showing 582 downregulated antisense transcripts (Fig. 2F). The divergence between those two sets of data needs to be further explored. We assume that downregulation of antisense transcripts is unlikely a direct effect by Set2, based on our results and previous studies [15, 16].

### Antisense transcripts do not affect the expression of the corresponding sense transcripts

**Fig. 3:**
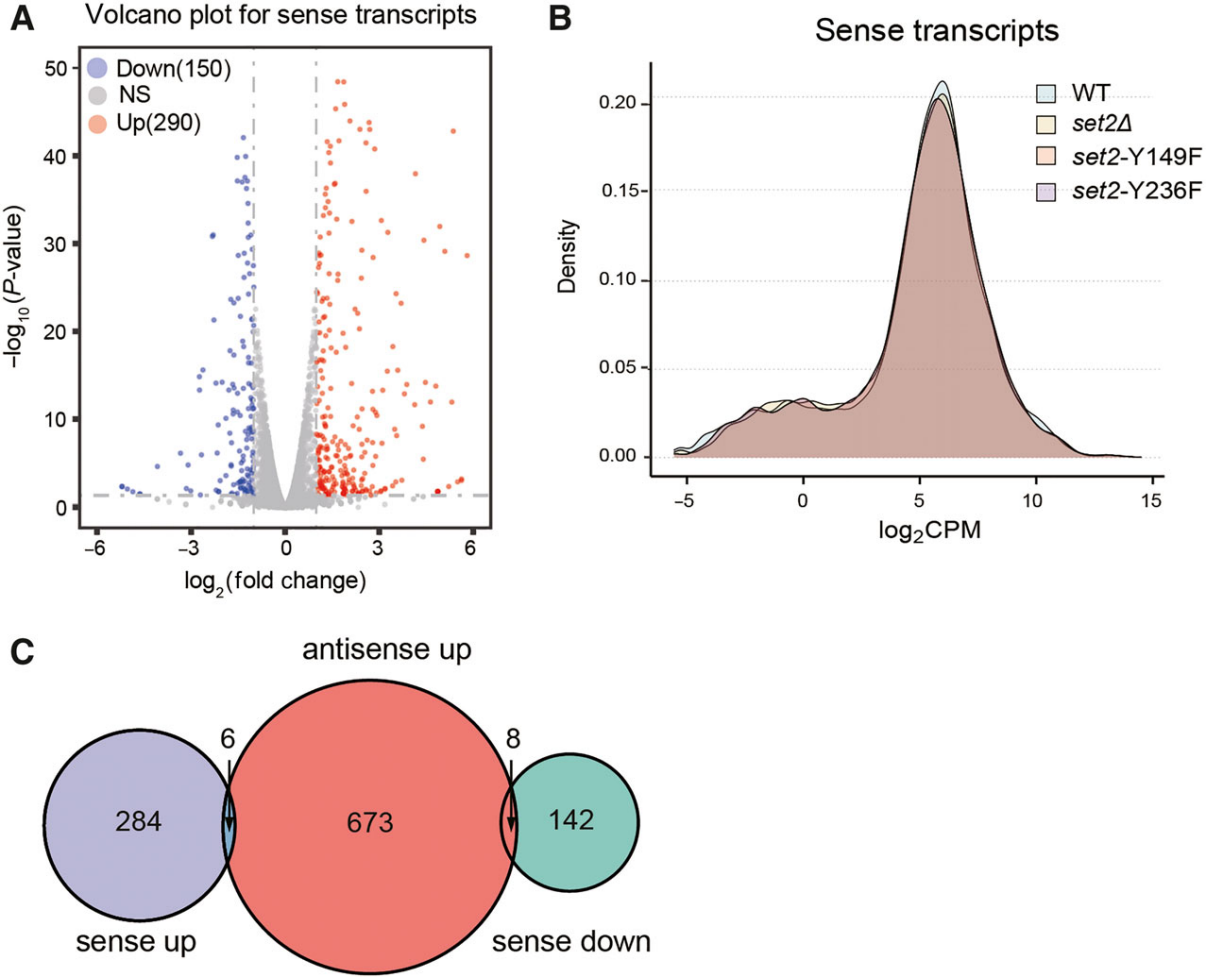
Antisense transcripts do not affect the expression levels of the sense genes. A, Volcano plot showing the expression pattern of sense genes upon deletion of *SET2*. Red dots represent significantly upregulated genes, and blue dots represent significantly downregulated genes. B, Density map showing the gene expression distribution in different mutant strains. Counts per million (CPM) were calculated in the edger software and used to measure gene expression. C, Venn diagram comparison between differentially expressed sense genes and antisense transcripts suppressed by Set2.

Previous studies have shown that certain antisense transcripts affect the transcription events of the corresponding sense genes [25]. Therefore, we wanted to explore whether SRATs could affect the expression of their corresponding sense genes. We noticed that 290 upregulated genes and 150 downregulated genes were identified in *set2*∆ cells relative to the WT (Fig. 3A). The gene density map showed that the gene expression patterns from sense transcripts were very similar between each indicated strain (Fig. 3B). Nevertheless, the altered expression changes of sense genes were not due to the production of corresponding antisense transcripts, as the majority of 687 genes with upregulated SRATs showed very few changes in sense gene expression (8 genes upregulated and 6 genes downregulated) in the *set2∆* strain (Fig. 3C). These data indicated that the expression of SRATs from the antisense strand generally does not affect sense transcription under nutrient‐rich conditions.

We observed the expression levels of a few genes affected by the production of antisense transcripts, in which six corresponding sense genes were upregulated accompanied by upregulation of the antisense transcript (Table 1). Interestingly, the proteins encoded by these genes are related to cell division, cell flocculation and energy metabolism. For example, Fig 2 protein is responsible for maintaining the integrity of the cell wall during cell mating to ensure high mating efficiency [26]. As a cell wall protein, Flo10 directly participates in adhesive cell–cell interactions in the process of flocculation [27]. Sfl1 is involved in cell surface assembly and the regulation of the flocculation process [28]. In addition, 8 corresponding sense genes were downregulated by the upregulated expression of antisense transcripts (Table 2). Proteins encoded by these genes are related to the process of cell mitosis and energy metabolism. For example, Clb6 can interact with Cdc28 to regulate the G1/S process during mitosis [29]. Glucose transporter encoded by *HXT2* plays an important role in energy metabolism [30]. These results suggested that antisense transcripts might cross talk with the sense strand to regulate gene expression through some unknown mechanism, which is of particular interest to be explored in the near future.

**Table 1:** Upregulated sense genes with the upregulation of antisense transcripts.

Gene name	log_2_FC	*P*‐value	Antisense transcripts	log_2_FC	*P*‐value
*FIG2*	1.22	6.47E−34	chrIII_268287_269650	6.08	1.25E−54
*FLO10*	1.26	2.11E−22	chrXI_646150_649088	4.35	4.98E−191
*GTT2*	1.08	1.94E−17	chrXII_21160_21867	2.33	1.90E−14
*HEF3*	1.37	1.66E−35	chrXIV_607466_608421	3.70	6.04E−36
*SFL1*	1.03	2.06E−14	chrXV_587861_589241	2.19	1.84E−24
*PCA1*	1.48	6.05E−51	chrII_792527_796498	4.41	1.33E−140

**Table 2:** Downregulated sense genes with the upregulation of antisense transcripts.

Gene name	log_2_FC	*P*‐value	Antisense transcripts	log_2_FC	*P*‐value
*AVT2*	−1.03	2.63E−14	chrV_30251_31501	2.97	2.74E−30
*ADE5,7*	−1.23	5.40E−37	chrVII_56171_57183	1.96	3.14E−10
*CLB6*	−1.04	6.77E−05	chrVII_706126_706667	2.60	8.40E−12
*CLB6*	−1.04	6.77E−05	chrVII_705368_706105	2.50	6.52E−15
*YJL213W*	−1.20	1.67E−28	chrX_31954_33105	2.25	8.46E−12
*SRY1*	−1.91	1.18E−67	chrXI_17659_18490	1.87	1.22E−14
*YLR124W*	−3.34	7.80E−07	chrXII_391411_393246	1.60	5.78E−27
*HXT2*	−1.07	1.81E−08	chrXIII_288228_289458	1.98	2.45E−14
*INP2*	−1.17	4.04E−17	chrXIII_584350_585354	2.83	6.67E−25

### Different H3K36 methylation states showed shared roles in repressing antisense transcription

**Fig. 4:**
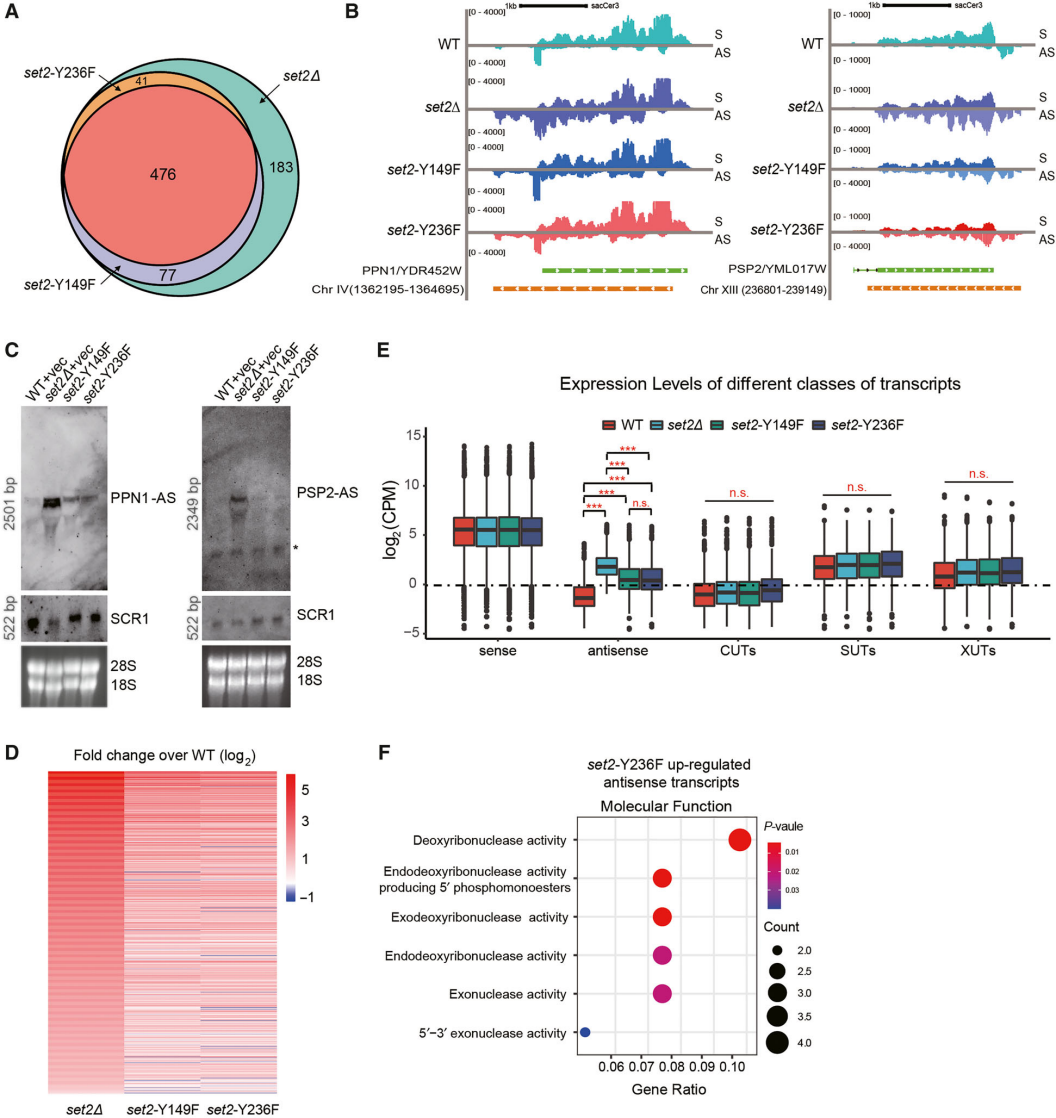
Shared roles of H3K36 methylation in the production of antisense transcripts. A, Venn diagram showing the overlap of statistically significant upregulated antisense transcripts produced upon *set2* deletion or the indicated Set2 mutants. B, Genome browser profile showing the distribution of transcripts produced in WT, *set2Δ*, *set2*‐Y149F and *set2*‐Y236F mutants over the *PPN1/YDR452W* and *PSP2/YML017W*. ChrIV (1362195‐1364695) and ChrXIII (236801‐239149) represent the antisense transcripts. The sense strand (S) on top runs from left to right, and the antisense strand (AS) on bottom runs from right to left. C, Strand‐specific northern blot probing for *PPN1*‐AS or *PSP2*‐AS using total RNA in the indicated strains. Probed *SCR1* and total RNA staining were used as loading controls. Asterisk represents nonspecific signals. The sizes of nucleotides were present. D, Heatmap showing the expression of antisense transcripts in different Set2 mutants over WT. E, Boxplot showing the abundance of the different classes of transcripts in the indicated strains. Expression levels of different classes of antisense transcripts were compared from 3 independent experiments (*n* = 3), and significance was calculated using the Wilcoxon test, ‘n.s.’, not significant; ***P < 0.001. F, GO analysis of specifically upregulated antisense transcripts ranking in the top 6 in *set2*‐Y236F mutants. An adjusted *P*‐value (< 0.05) was used for term ranking and selection.

Next, we attempted to determine the expression differences of antisense transcripts in the *set2*‐Y149F and *set2*‐Y236F mutants. Applying the same cut‐off as described earlier, we obtained 553 upregulated antisense transcripts in *set2*‐Y149F, which represents H3K36me3, and 517 upregulated antisense transcripts in *set2*‐Y236F, which represents H3K36me1/2 (Fig. 4A). A total of 476 antisense transcripts were upregulated in both mutant strains, and the overlap between them reached over 90% (Fig. 4A). Genome browser profiles indicated that the antisense transcripts produced at the loci of *YDR452W* and *YML017W* showed robust enrichment in the *set2Δ* strain and moderate abundance in the *set2*‐Y149F and *set2*‐Y236F mutants, while very low antisense expression was observed in coding regions of the WT strain (Fig. 4B). We validated the strand‐specific RNA‐seq experiments by northern blotting, which showed a similar result, underlining the reproducible production of the SRATs in different mutants (Fig. 4C). Pearson’s correlation analysis of the antisense transcript expression in replicated samples indicated a stronger correlation of the two mutants than that of the *set2∆* or WT strain (Fig. S2). Moreover, each individual upregulated antisense transcript identified in the two mutant strains displayed a comparable expression level, which was much lower than the expression level of the *set2Δ* mutants (Fig. 4D). All of these data suggest that different H3K36 methylation states redundantly contribute to the repression of antisense transcripts.

Previous studies have identified different classes of cryptic transcripts, such as cryptic unstable transcripts (CUTs) [4], Xrn1‐sensitive unstable transcripts (XUTs) [6] and stable unannotated transcripts (SUTs) [6], by NET‐seq in *Saccharomyces cerevisiae*. We wanted to further investigate whether different H3K36 methylation states exert unique regulation of such cryptic transcripts. However, we found that deletion of *SET2* or H3K36 methyl‐deficient mutants did not affect the production of either sense transcripts or these cryptic transcripts (Fig. 4E). Once again, we observed that the loss of a specific methylation state caused upregulated expression of antisense transcripts, but deletion of *SET2* resulted in a higher level of antisense transcription, which reinforced the coordinated roles of H3K36me1/me2 and H3K36me3 in the repression of antisense transcripts (Fig. 4E).

To explore the potential regulatory roles of upregulated antisense transcripts upon loss of Set2 or H3K36 dimethylation or trimethylation, GO analysis was utilized. The antisense transcripts upon loss of Set2 were mainly correlated with cell vacuoles, cell division and budding positions (Fig. S3). Interestingly, the top 10 sense genes in both cellular component enrichment and biological function processes were consistent (Fig. S3). However, the results demonstrated that H3K36me3 might mediate unique biological roles. We found that the 77 upregulated antisense transcripts uniquely identified in the *set2*‐Y149F strain were not enriched in any biological process or cellular component, whereas the 41 upregulated antisense transcripts uniquely identified in the *set2*‐Y236F strain predominantly participated in various kinase activities that are required for DNA replication and DNA damage repair (Fig. 4F). Altogether, these results implied that such antisense transcripts may directly or indirectly participate in these biological processes, and the real functions need to be verified in future studies.

## Discussion

In this study, we took advantage of a different bioinformatics method to explore a class of antisense transcripts suppressed by H3K36 in different methylation states. Using this method to filter antisense reads in the whole genome, we were able to identify more antisense transcripts in the Set2 null mutant than in a previous study [15]. Moreover, we showed that H3K36me1/2 mediated by the *set2*‐Y236F mutant or H3K36me3 mediated by the *set2*‐Y149F mutant is not unique for regulating the expression of antisense transcripts, which illustrates a coregulatory mechanism of different H3K36 methylation states under nutrient‐rich conditions. A recent study by DiFiore et al. also showed a shared role of different H3K36 methylation states on antisense transcription upon nutrient starvation [16]. Using a published data obtained from the same laboratory, in which there are 439 genes with bidirectional cryptic transcripts in *set2∆* cells upon nutrient deprivation, they re‐evaluated the location with cryptic initiation sites (CISs) in *set2* mutants bearing H3K36me1/2 or H3K36me3 alone[31]. Different from that they validated production of the CIS position in these mutants using a yeast reporter system [31], our genome‐wide analysis provided much larger numbers of antisense transcripts (3663 ATE genes) than those from their data (Fig. 2A). Moreover, we also focused on the final production of antisense transcripts and compared the different expression levels of antisense transcripts in different H3K36 methylation states directly by strand‐specific northern blot analysis (Fig. 4C). Therefore, we argue that our analysis provides an incremental contribution towards antisense transcription.

Meanwhile, we devoted more attention to the functions of the sense genes corresponding to these antisense transcripts under nutrient‐rich conditions. It has long been reported that Set2‐mediated H3K36 methylation is involved in numerous DNA damage repair processes, development and ageing. Interestingly, we found that the antisense transcripts regulated by H3K36 methylation were also enriched in the process of DNA replication, mitosis or cell budding (Tables 1 and 2). GO enrichment analysis showed that the corresponding sense genes with upregulated antisense transcripts in cells lacking H3K36me3 were mainly enriched with endodeoxyribonucleases or exonucleases. These enzymes are often involved in DNA replication or DNA damage repair pathways [32]. Given that upregulated antisense transcripts do not affect the expression of the corresponding sense transcripts (Fig. 2), it is intriguing that how these antisense transcripts exert biological functions? A line of evidence suggests that antisense transcripts might regulate the sense genes during the post‐transcriptional process [33]. For example, antisense expression controls translational efficiency by affecting the produced transcript isoform of the zinc‐finger E‐box‐binding homeobox 2 gene (*ZEB2*), which encodes a transcriptional repressor of E‐cadherin [34]. In addition, an antisense transcript regulates the translational efficiency of the ubiquitin carboxy‐terminal hydrolase L1 gene (*Uchl1*) [35]. In bacteria, the antisense transcript *SymR* can directly bind to the 5’ end of the *SymE* transcript, which inhibits *SymE* translation [36]. Alternatively, antisense transcription may affect sense gene expression during transcriptional and cotranscriptional processes under certain stress conditions [33]. For instance, accumulated antisense transcripts in old yeast cells were exhibited in a subset of genes and are detrimental to life span, which suggests that ageing‐related genes might be affected under a stress condition [37]. It would be interesting to examine the biological relevance of antisense transcription with corresponding sense expression using those *set2* mutants under various stimulus conditions. Although the information we obtained here is only the tip of the iceberg for the mechanism by which H3K36 methylation inhibits antisense transcripts, we believe that this evidence may provide a new tool for characterizing other unique biological functions of different H3K36 methylation states.

## Conflict of interest

The authors declare no conflict of interest.

## Author contributions

HND and YCM conceived and designed the project. YCM, JF, YML and YL performed experiments. FH provided reagents. YCM, HND, FL and YC analysed and interpreted the data. YCM and HND wrote the paper.

## Supporting information

Fig. S1. Comparison of the SET domains between yeast Set2 protein and human SETD2 protein. A, Sequence alignment of the SET domains of yeast Set2 protein (a.a. 1–300) and human SETD2 protein (a.a. 1447–1701). The secondary structures were displayed using ESPript 3.0 software. α1, α2: α‐helices. η1–η3: 3_10_‐helices; β1–β8: β‐sheet; TT: β‐turns: TTT: α‐turns. B, Structural comparison of the SET domains between human SETD2 (green) and Set2 protein (red).
Fig. S2. Heatmap showing the correlation of antisense expression between WT and the indicated Set2 strains. The numbers show the Pearson correlation coefficient between each pair of samples.
Fig. S3. GO analysis of the enriched sense genes ranking in the tops of biological function processes shown in (A) or cellular component enrichment shown in (B) regulated by upregulated antisense transcripts upon deletion of *SET2*.

## Data Availability

Raw RNA‐seq data have been deposited in the GEO database with accession number GSE167338. Additional data will be available from the corresponding author upon reasonable request.

## References

[1] Wagner EG and Simons RW (1994) Antisense RNA control in bacteria, phages, and plasmids. Annu Rev Microbiol 48, 713–742. 7826024 10.1146/annurev.mi.48.100194.003433

[2] Vanhée‐Brossollet C and Vaquero C (1998) Do natural antisense transcripts make sense in eukaryotes? Gene 211, 1–9. 9573333 10.1016/s0378-1119(98)00093-6

[3] Ozsolak F , Kapranov P , Foissac S , Kim SW , Fishilevich E , Monaghan AP , John B and Milos PM (2010) Comprehensive polyadenylation site maps in yeast and human reveal pervasive alternative polyadenylation. Cell 143, 1018–1029. 21145465 10.1016/j.cell.2010.11.020 PMC3022516

[4] Neil H , Malabat C , d'Aubenton‐Carafa Y , Xu Z , Steinmetz LM and Jacquier A (2009) Widespread bidirectional promoters are the major source of cryptic transcripts in yeast. Nature 457, 1038–1042. 19169244 10.1038/nature07747

[5] Xu Z , Wei W , Gagneur J , Perocchi F , Clauder‐Münster S , Camblong J , Guffanti E , Stutz F , Huber W and Steinmetz LM (2009) Bidirectional promoters generate pervasive transcription in yeast. Nature 457, 1033–1037. 19169243 10.1038/nature07728 PMC2766638

[6] van Dijk EL , Cl C , d’Aubenton‐Carafa Y , Gourvennec S , Kwapisz M , Roche V , Bertrand C , Silvain M , Legoix‐Né P , Loeillet S *et al*. (2011) XUTs are a class of Xrn1‐sensitive antisense regulatory non‐coding RNA in yeast. Nature 475, 114–117. 21697827 10.1038/nature10118

[7] McDaniel SL and Strahl BD (2017) Shaping the cellular landscape with Set2/SETD2 methylation. Cell Mol Life Sci 74, 3317–3334. 28386724 10.1007/s00018-017-2517-x PMC5545052

[8] Venkatesh S , Smolle M , Li H , Gogol MM , Saint M , Kumar S , Natarajan K and Workman JL (2012) Set2 methylation of histone H3 lysine 36 suppresses histone exchange on transcribed genes. Nature 489, 452–455. 22914091 10.1038/nature11326

[9] DeGennaro CM , Alver BH , Marguerat S , Stepanova E , Davis CP , Bahler J , Park PJ and Winston F (2013) Spt6 regulates intragenic and antisense transcription, nucleosome positioning, and histone modifications genome‐wide in fission yeast. Mol Cell Biol 33, 4779–4792. 24100010 10.1128/MCB.01068-13 PMC3889546

[10] Cheung V , Chua G , Batada NN , Landry CR , Michnick SW , Hughes TR and Winston F (2008) Chromatin‐ and transcription‐related factors repress transcription from within coding regions throughout the Saccharomyces cerevisiae genome. PLoS Biol 6, e277. 18998772 10.1371/journal.pbio.0060277 PMC2581627

[11] Kaplan CD , Laprade L and Winston F (2003) Transcription elongation factors repress transcription initiation from cryptic sites. Science 301, 1096–1099. 12934008 10.1126/science.1087374

[12] Smolle M , Venkatesh S , Gogol MM , Li H , Zhang Y , Florens L , Washburn MP and Workman JL (2012) Chromatin remodelers Isw1 and Chd1 maintain chromatin structure during transcription by preventing histone exchange. Nat Struct Mol Biol 19, 884–892. 22922743 10.1038/nsmb.2312 PMC3560298

[13] Lee CH , Wu J and Li B (2013) Chromatin remodelers fine‐tune H3K36me‐directed deacetylation of neighbor nucleosomes by Rpd3S. Mol Cell 52, 255–263. 24055344 10.1016/j.molcel.2013.08.024 PMC3825818

[14] Carrozza MJ , Li B , Florens L , Suganuma T , Swanson SK , Lee KK , Shia W‐J , Anderson S , Yates J , Washburn MP *et al*. (2005) Histone H3 methylation by Set2 directs deacetylation of coding regions by Rpd3S to suppress spurious intragenic transcription. Cell 123, 581–592. 16286007 10.1016/j.cell.2005.10.023

[15] Venkatesh S , Li H , Gogol MM and Workman JL (2016) Selective suppression of antisense transcription by Set2‐mediated H3K36 methylation. Nat Commun 7, 13610. 27892455 10.1038/ncomms13610 PMC5133703

[16] DiFiore JV , Ptacek TS , Wang Y , Li B , Simon JM and Strahl BD (2020) Unique and Shared Roles for Histone H3K36 Methylation States in Transcription Regulation Functions. Cell Rep 31, 107751. 32521276 10.1016/j.celrep.2020.107751 PMC7334899

[17] Du HN , Fingerman IM and Briggs SD (2008) Histone H3 K36 methylation is mediated by a trans‐histone methylation pathway involving an interaction between Set2 and histone H4. Genes Dev 22, 2786–2798. 18923077 10.1101/gad.1700008 PMC2569878

[18] Zhang Y (2008) I‐TASSER server for protein 3D structure prediction. BMC Bioinformatics 9, 40. 18215316 10.1186/1471-2105-9-40 PMC2245901

[19] Yang S , Zheng X , Lu C , Li GM , Allis CD and Li H (2016) Molecular basis for oncohistone H3 recognition by SETD2 methyltransferase. Genes Dev 30, 1611–1616. 27474439 10.1101/gad.284323.116 PMC4973290

[20] Hao R , He J , Liu X , Gao G , Liu D , Cui L , Yu G , Yu W , Chen Y and Guo D (2015) Inhibition of hepatitis B virus gene expression and replication by hepatocyte nuclear factor 6. J Virol 89, 4345–4355. 25653429 10.1128/JVI.03094-14 PMC4442367

[21] Xiao B , Jing C , Wilson JR , Walker PA , Vasisht N , Kelly G , Howell S , Taylor IA , Blackburn GM and Gamblin SJ (2003) Structure and catalytic mechanism of the human histone methyltransferase SET7/9. Nature 421, 652–656. 12540855 10.1038/nature01378

[22] Collins RE , Tachibana M , Tamaru H , Smith KM , Jia D , Zhang X , Selker EU , Shinkai Y and Cheng X (2005) In vitro and in vivo analyses of a Phe/Tyr switch controlling product specificity of histone lysine methyltransferases. J Biol Chem 280, 5563–5570. 15590646 10.1074/jbc.M410483200 PMC2696276

[23] Cheng X , Collins RE and Zhang X (2005) Structural and sequence motifs of protein (histone) methylation enzymes. Annu Rev Biophys Biomol Struct 34, 267–294. 15869391 10.1146/annurev.biophys.34.040204.144452 PMC2733851

[24] Herz HM , Garruss A and Shilatifard A (2013) SET for life: biochemical activities and biological functions of SET domain‐containing proteins. Trends Biochem Sci 38, 621–639. 24148750 10.1016/j.tibs.2013.09.004 PMC3941473

[25] Xu Z , Wei W , Gagneur J , Clauder‐Munster S , Smolik M , Huber W and Steinmetz LM (2011) Antisense expression increases gene expression variability and locus interdependency. Mol Syst Biol 7, 468. 21326235 10.1038/msb.2011.1 PMC3063692

[26] Zhang M , Bennett D and Erdman SE (2002) Maintenance of mating cell integrity requires the adhesin Fig2p. Eukaryot Cell 1, 811–822. 12455698 10.1128/EC.1.5.811-822.2002 PMC126742

[27] He LY , Zhao XQ , Ge XM and Bai FW (2012) Identification and functional study of a new FLO10‐derivative gene from the industrial flocculating yeast SPSC01. J Ind Microbiol Biotechnol 39, 1135–1140. 22466447 10.1007/s10295-012-1121-1

[28] Fujita A , Kikuchi Y , Kuhara S , Misumi Y , Matsumoto S and Kobayashi H (1989) Domains of the SFL1 protein of yeasts are homologous to Myc oncoproteins or yeast heat‐shock transcription factor. Gene 85, 321–328. 2697640 10.1016/0378-1119(89)90424-1

[29] Mendenhall MD and Hodge AE (1998) Regulation of Cdc28 cyclin‐dependent protein kinase activity during the cell cycle of the yeast Saccharomyces cerevisiae. Microbiol Mol Biol Rev 62, 1191–1243. 9841670 10.1128/mmbr.62.4.1191-1243.1998 PMC98944

[30] Kasahara T , Maeda M , Ishiguro M and Kasahara M (2007) Identification by comprehensive chimeric analysis of a key residue responsible for high affinity glucose transport by yeast HXT2. J Biol Chem 282, 13146–13150. 17369259 10.1074/jbc.C700041200

[31] McDaniel SL , Hepperla AJ , Huang J , Dronamraju R , Adams AT , Kulkarni VG , Davis IJ and Strahl BD (2017) H3K36 Methylation Regulates Nutrient Stress Response in Saccharomyces cerevisiae by Enforcing Transcriptional Fidelity. Cell Rep 19, 2371–2382. 28614721 10.1016/j.celrep.2017.05.057 PMC5528882

[32] Trachuk LA , Bushueva AM , Shevelev AB , Novgorodova SA , Akparov V and Chestukhina GG (2002) Characterization of S1' subsite specificity of Thermoactinomyces vulgaris carboxypeptidase T by site‐directed mutagenesis. Vopr Med Khim 48, 577–585. 12698557

[33] Pelechano V and Steinmetz LM (2013) Gene regulation by antisense transcription. Nat Rev Genet 14, 880–893. 24217315 10.1038/nrg3594

[34] Beltran M , Puig I , Pena C , Garcia JM , Alvarez AB , Pena R , Bonilla F and de Herreros AG (2008) A natural antisense transcript regulates Zeb2/Sip1 gene expression during Snail1‐induced epithelial‐mesenchymal transition. Genes Dev 22, 756–769. 18347095 10.1101/gad.455708 PMC2275429

[35] Carrieri C , Cimatti L , Biagioli M , Beugnet A , Zucchelli S , Fedele S , Pesce E , Ferrer I , Collavin L , Santoro C *et al*. (2012) Long non‐coding antisense RNA controls Uchl1 translation through an embedded SINEB2 repeat. Nature 491, 454–457. 23064229 10.1038/nature11508

[36] Kawano M , Aravind L and Storz G (2007) An antisense RNA controls synthesis of an SOS‐induced toxin evolved from an antitoxin. Mol Microbiol 64, 738–754. 17462020 10.1111/j.1365-2958.2007.05688.x PMC1891008

[37] Sen P , Dang W , Donahue G , Dai J , Dorsey J , Cao X , Liu W , Cao K , Perry R , Lee JY *et al*. (2015) H3K36 methylation promotes longevity by enhancing transcriptional fidelity. Genes Dev 29, 1362–1376. 26159996 10.1101/gad.263707.115 PMC4511212

