# Supplementary material for: Set2‐mediated H3K36 methylation states redundantly repress the production of antisense transcripts: role in transcription regulation
Source: FEBS Open Bio. 2021 Jun 28;11(8):2225–35. doi: 10.1002/2211-5463.13226 (PMC8329787; doi:10.1002/2211-5463.13226)
Supplement: Supplementary file 1 — Fig. S1. Comparison of the SET domains between yeast Set2 protein and human SETD2 protein. A, Sequence alignment of the SET domains of yeast Set2 protein (a.a. 1–300) and human SETD2 protein (a.a. 1447–1701). The secondary structures were displayed using ESPript 3.0 software. α1, α2: α‐helices. η1–η3: 310‐helices; β1–β8: β‐sheet; TT: β‐turns: TTT: α‐turns. B, Structural comparison of the SET domains between human SETD2 (green) and Set2 protein (red). Fig. S2. Heatmap showing the correlation of antisense expression between WT and the indicated Set2 strains. The numbers show the Pearson correlation coefficient between each pair of samples. Fig. S3. GO analysis of the enriched sense genes ranking in the tops of biological function processes shown in (A) or cellular component enrichment shown in (B) regulated by upregulated antisense transcripts upon deletion of SET2. [file FEB4-11-2225-s001.pdf]

Supplemental Figure legends

Fig. S1. Comparison of the SET domains between yeast Set2 protein and human SETD2 protein. **A** Sequence alignment of the SET domains of yeast Set2 protein (a.a. 1-300) and human SETD2 protein (a.a. 1447-1701). The secondary structures were displayed using ESPript 3.0 software. α1, α2: α-helices. η1-η3: 3_{10}-helices; β1-β8: β-sheet; TT: β-turns: TTT: α-turns. **B** Structural comparison of the SET domains between human SETD2 (green) and Set2 protein (red).

Fig. S2. Heatmap showing the correlation of antisense expression between WT and the indicated Set2 strains. The numbers show the Pearson correlation coefficient between each pair of samples.

Fig. S3. GO analysis of the enriched sense genes ranking in the tops of biological function processes shown in **A** or cellular component enrichment shown in **B** regulated by up-regulated antisense transcripts upon deletion of *SET2*.

Figure S1

A

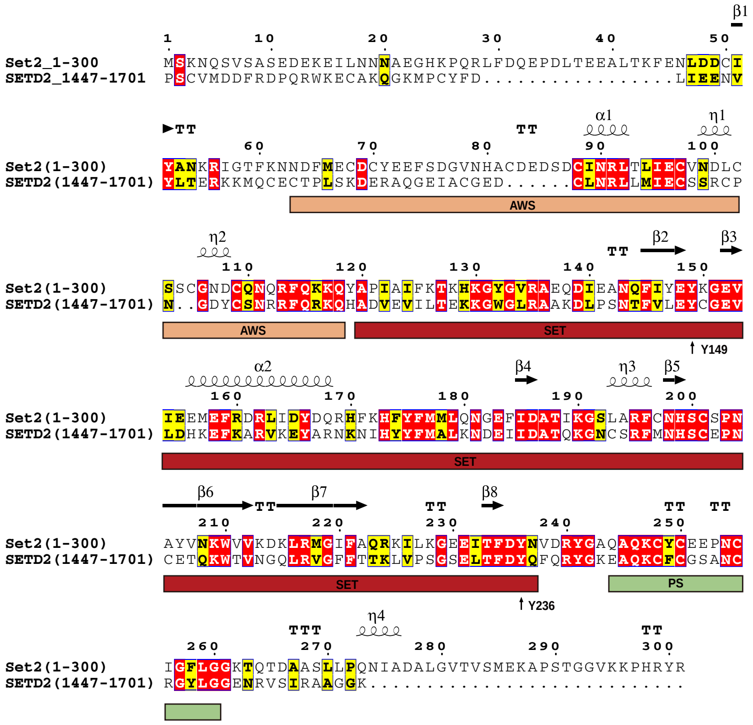

B

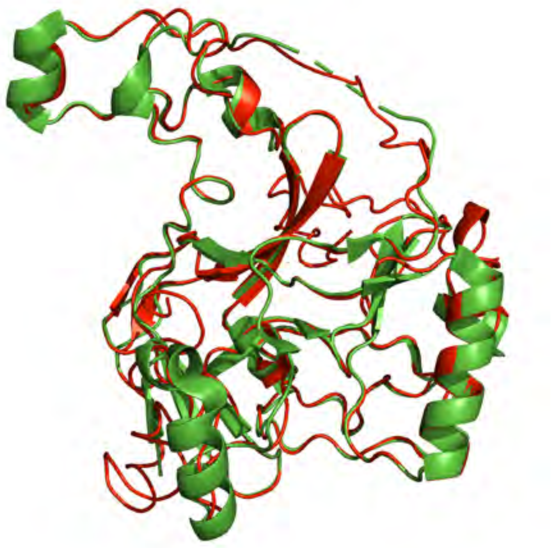

Green: human SET domain (1447-1701 a.a.)
Red: yeast SET domain (1-300 a.a.)

Figure S2

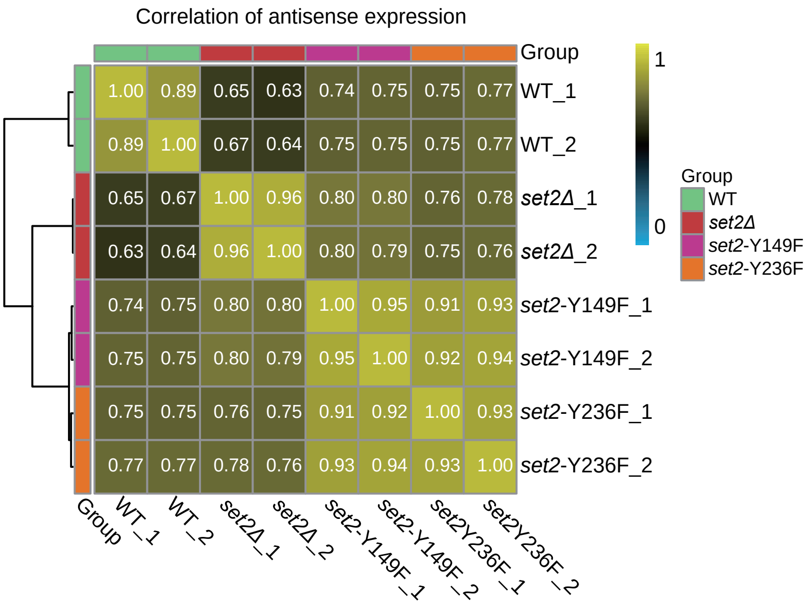

Correlation of antisense expression
Group
1.00 0.89 0.65 0.63 0.74 0.75 0.75 0.77 WT_1
0.89 1.00 0.67 0.64 0.75 0.75 0.75 0.77 WT_2
0.65 0.67 1.00 0.96 0.80 0.80 0.76 0.78 set2Δ_1
0.63 0.64 0.96 1.00 0.80 0.79 0.75 0.76 set2Δ_2
0.74 0.75 0.80 0.80 1.00 0.95 0.91 0.93 set2-Y149F_1
0.75 0.75 0.80 0.79 0.95 1.00 0.92 0.94 set2-Y149F_2
0.75 0.75 0.76 0.75 0.91 0.92 1.00 0.93 set2-Y236F_1
0.77 0.77 0.78 0.76 0.93 0.94 0.93 1.00 set2-Y236F_2
Group
WT_1
WT_2
set2Δ_1
set2Δ_2
set2-Y149F_1
set2-Y149F_2
set2Y236F_1
set2Y236F_2
1
0
Group
WT
set2Δ
set2-Y149F
set2-Y236F

Figure S3

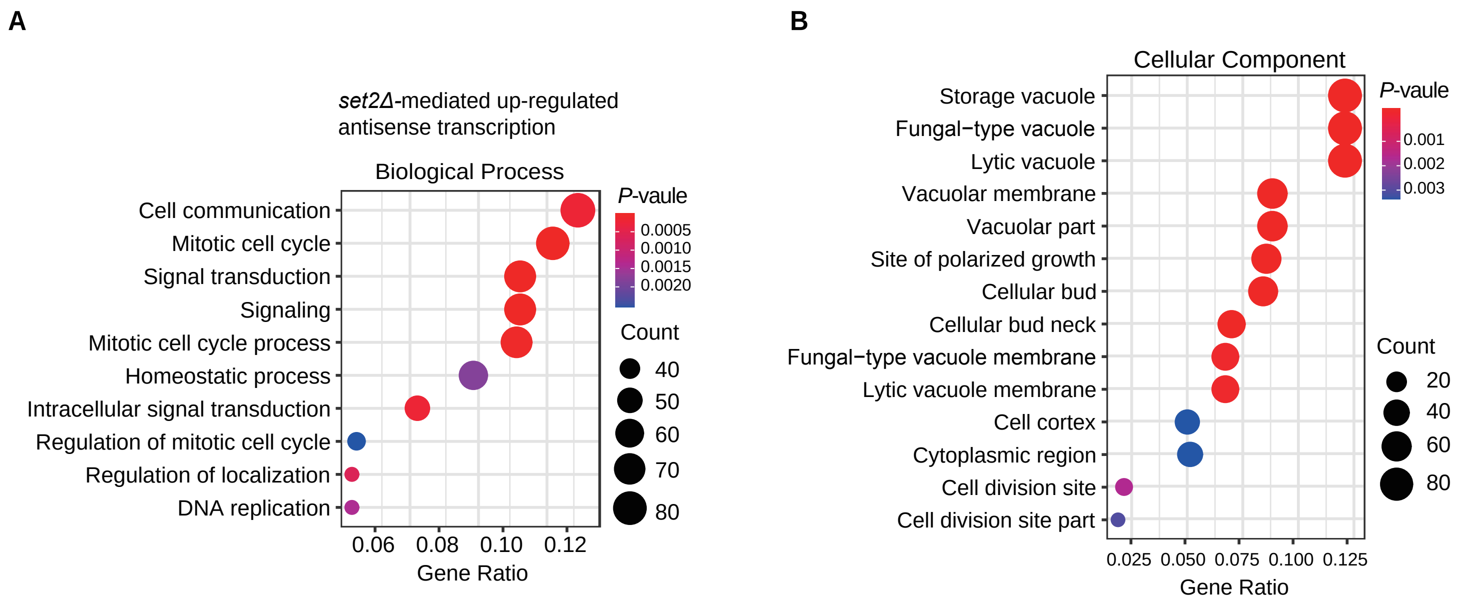
A
set2Δ-mediated up-regulated antisense transcription
Biological Process
Cell communication
Mitotic cell cycle
Signal transduction
Signaling
Mitotic cell cycle process
Homeostatic process
Intracellular signal transduction
Regulation of mitotic cell cycle
Regulation of localization
DNA replication
0.06 0.08 0.10 0.12
Gene Ratio
P-vaule
0.0005
0.0010
0.0015
0.0020
Count
40
50
60
70
80
B
Cellular Component
Storage vacuole
Fungal−type vacuole
Lytic vacuole
Vacuolar membrane
Vacuolar part
Site of polarized growth
Cellular bud
Cellular bud neck
Fungal−type vacuole membrane
Lytic vacuole membrane
Cell cortex
Cytoplasmic region
Cell division site
Cell division site part
0.025 0.050 0.075 0.100 0.125
Gene Ratio
P-vaule
0.001
0.002
0.003
Count
20
40
60
80
